# Response to the letter to the editor regarding “safety of tenecteplase versus alteplase for intravenous thrombolysis in acute ischemic stroke patients with direct oral anticoagulation”

**DOI:** 10.1186/s42466-026-00459-7

**Published:** 2026-02-09

**Authors:** Lena Mers, Stefan T. Gerner

**Affiliations:** 1 Department of Neurology, University Hospital Erlangen, Schwabachanlage 6, Erlangen, 91054 Germany; 2 Department of Neurology, University Hospital Giessen, Klinikstraße 33, 35392 Giessen, Germany

Dear editor, dear colleagues,

We thank Zupan et al. for their thoughtful and constructive comments on our recent publication evaluating the safety of tenecteplase compared with alteplase for intravenous thrombolysis in acute ischemic stroke patients with recent direct oral anticoagulant (DOAC) intake. We appreciate their careful appraisal of our findings and their recognition of the clinical relevance of this topic in an era of steadily increasing DOAC use.

We agree with the authors that evidence guiding intravenous thrombolysis in DOAC-treated patients remains limited and is largely based on observational data prone to potential bias [1]. In this context, our study aimed to contribute real-world safety data, particularly regarding tenecteplase, which is increasingly used in routine stroke care but remains underrepresented in this specific population. As highlighted, our analysis did not demonstrate an increased risk of symptomatic intracranial hemorrhage, overall hemorrhagic complications, or mortality with tenecteplase compared with alteplase, while acknowledging the inherent limitations of a retrospective, single-center design and modest sample size [2].

We appreciate the considerations regarding thrombus composition and fibrin architecture under DOAC exposure represent an interesting biological framework for understanding thrombolytic response. Experimental data suggesting structural differences in clots formed under anticoagulation, as well as differential fibrin specificity between thrombolytic agents, may indeed help generate hypotheses for future translational and clinical studies [3]. However, we would like to emphasize that, from a clinical standpoint, these mechanistic considerations remain secondary to a more fundamental and currently unresolved question.

Specifically, the primary clinical issue is whether intravenous thrombolysis can be performed safely in patients with recent DOAC intake, irrespective of the thrombolytic agent used and time-point of last intake. This question is of major practical relevance, as clarification of safety alone has the potential to expand access to reperfusion therapy for a substantial number of patients who are currently excluded or treated with uncertainty [4]. In this context, our study was primarily designed to contribute real-world safety data and not to establish superiority or differential efficacy between tenecteplase and alteplase.

Our findings suggest that, within the limitations of a retrospective single-center analysis, intravenous thrombolysis in carefully selected DOAC-treated patients was not associated with an excessive risk of symptomatic intracranial hemorrhage or overall hemorrhagic complications, and that tenecteplase did not demonstrate an unfavorable safety profile compared with alteplase [2]. Any observations regarding potential differences between thrombolytic agents should therefore be interpreted as exploratory and hypothesis-generating rather than as evidence of differential efficacy.

We concur with Zupan et al. that prospective, multicenter investigations are required to systematically address both safety and efficacy of intravenous thrombolysis in DOAC-treated patients. Such studies should first aim to establish robust safety profiles, ideally incorporating predefined clinical and imaging endpoints to minimize bias that currently limits this field of research. Only once safety is clearly established can secondary questions — such as potential agent-specific effects or interactions with thrombus composition — be meaningfully evaluated. The ongoing ACT-GLOBAL Thrombolysis Domain (ClinicalTrials.gov ID: NCT06352632), PASSION (EU trial number: 2025-521762-99), and DO-IT (ClinicalTrials.gov ID: NCT06571149) trials represent important steps in this direction.

In summary, we thank the authors for their insightful commentary. While mechanistic hypotheses regarding thrombus characteristics and fibrin specificity are intriguing, we believe that ensuring the safe application of intravenous thrombolysis in DOAC-treated patients remains the most pressing clinical priority. We hope that ongoing and future studies will help refine patient selection and ultimately improve access to effective reperfusion therapies in this growing patient population.

Sincerely,

Lena Mers and Stefan T. Gerner

## Data Availability

Not applicable.
